# Vitamin C deficiency mimicking inflammatory bone disease of the hand

**DOI:** 10.1186/s12969-020-00439-4

**Published:** 2020-06-09

**Authors:** Emily J. Liebling, Raymond W. Sze, Edward M. Behrens

**Affiliations:** 1 Division of Rheumatology, The Children’s Hospital of Philadelphia, 3401 Civic Center Blvd, Philadelphia, PA 19104 USA; 2 Department of Radiology, The Children’s Hospital of Philadelphia, Philadelphia, PA USA; 3 Perelman School of Medicine of the University of Pennsylvania, Philadelphia, PA USA

**Keywords:** Vitamin C deficiency, Inflammatory bone disease, Hand lesions

## Abstract

**Background:**

Severe vitamin C deficiency, or scurvy, encompasses a syndrome of multisystem abnormalities due to defective collagen synthesis and antioxidative functions. Among the more common presentations is a combination of oral or subcutaneous hemorrhage with lower extremity pain, the latter often exhibiting inflammatory bone changes on magnetic resonance imaging (MRI).

**Case presentation:**

A 12-year-old male with anorexia nervosa presented with asymmetric painful swelling of multiple fingers of both hands. Imaging demonstrated soft tissue and bone marrow edema of several phalanges, without arthritis, concerning for an inflammatory process. Extensive imaging and laboratory evaluations were largely unrevealing, with the exception of a severely low vitamin C level and a moderately low vitamin D level. A diagnosis of scurvy was made and supplementation was initiated. Within 3 weeks of treatment, serum levels of both vitamins normalized and the digital abnormalities resolved on physical exam.

**Conclusions:**

This represents the first description of scurvy manifesting with bone and soft tissue changes limited to the hands. There must be a high index of suspicion for scurvy in children with restricted dietary intake or malabsorption who have bone pain, irrespective of location of the lesions.

## Background

Scurvy, the clinical syndrome of vitamin C deficiency, has long been considered a disease of the past, limited to sailors and other individuals with little access to ascorbic acid-containing fruits and vegetables. While rare in the modern era, severe vitamin C deficiency continues to occasionally occur, and has been particularly reported in children with neurodevelopmental restrictive eating habits [1]. Vitamin C is an essential nutrient that has many functions, among which are its roles in collagen synthesis and multisystem antioxidative processes [2]. Low levels can, therefore, manifest as a pleiomorphic syndrome affecting the skin and hair, oral mucosa, musculoskeletal system, central nervous system, and bone marrow.

Published case reports of vitamin C deficiency describe children who present with leg pain and refusal to bear weight [3, 4]. Many have magnetic resonance imaging (MRI) findings of bilateral and symmetric bone marrow edema with periosteal and soft tissue signal abnormalities that are suspicious for infectious osteomyelitis, noninfectious osteomyelitis, or malignancy [5]. Here we describe a unique case of bone and soft tissue change secondary to vitamin C deficiency, which did not present with the classical pattern of lower extremity symptoms, but rather with multifocal finger pain and swelling.

## Case presentation

**Fig. 1:**
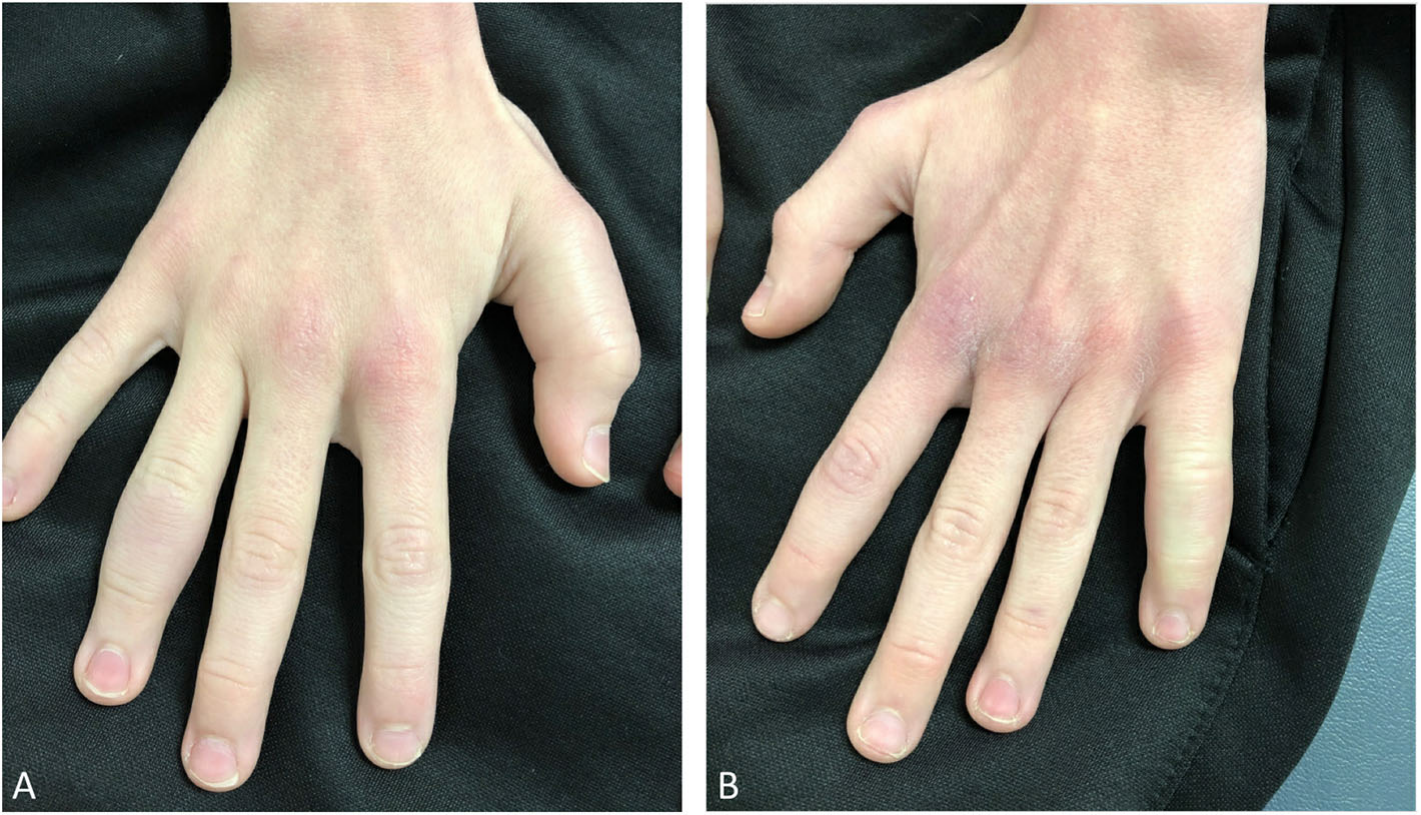
**a**. Swelling of right first proximal phalanx and fourth middle phalanx. **b**. Swelling of left fifth middle phalanx

A 12-year-old boy with anorexia nervosa presented with subacute multifocal digital swelling. He had been diagnosed with an eating disorder about 4 months prior to symptom onset, requiring admission to the Adolescent Medicine service for malnutrition, orthostasis and bradycardia, which resolved with monitored feeding. Despite ongoing behavioral therapy following discharge, however, adequate intake with continued weight gain remained a challenge. Over the course of 6 weeks, he developed additive and persistent swelling of the right hand first and fourth digits, followed by the left fifth digit (Fig. 1). The swelling was painful and unresponsive to nonsteroidal antiinflammatory drugs, there was no fever, and he identified no preceding trauma or illness. Physical exam was notable for fusiform swelling limited to the phalanges, as the joints themselves did not exhibit any discrete effusions. Furthermore, the overlying skin was normal in appearance and texture, without petechiae or ecchymoses, and no other mucocutaneous changes were present elsewhere. Complete musculoskeletal exam was otherwise normal, without other areas of swelling or pain. The patient appeared generally cachectic and review of the growth curve revealed significant weight loss with concomitant stunted vertical growth over the previous 2 years, with a sustained body mass index below the first percentile for age. He had previously exhibited a normal fecal calprotectin and at the time of this presentation did not report any gastrointestinal abnormalities concerning for enterocolonic inflammation or malabsorption. He denied practicing restrictive eating behaviors, but his diet included few fruits and vegetables. He was then admitted for a comprehensive medical work up of this constellation of symptoms.

**Fig. 2:**
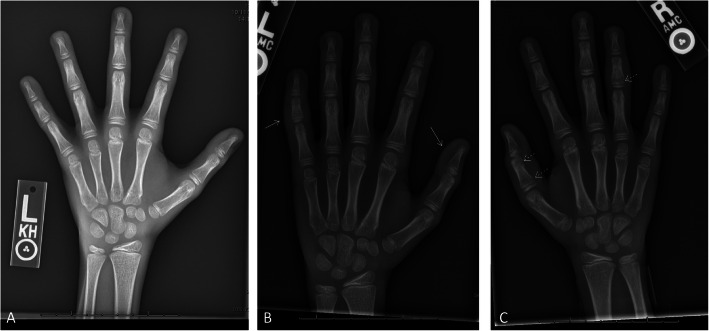
**a**. Left hand radiograph taken 6 months prior shows delayed bone age, but no other abnormalities of the bones or soft tissues. Shown here for comparison. **b**. Left hand radiograph at presentation shows soft tissue swelling of the first and fifth digits (solid arrows). **c**. Right hand radiograph hand shows cortical erosion of the head of the first proximal phalanx and metaphyseal base of the fourth middle phalanx with periosteal reaction and overlying soft tissue swelling (dotted arrows)

**Fig. 3:**
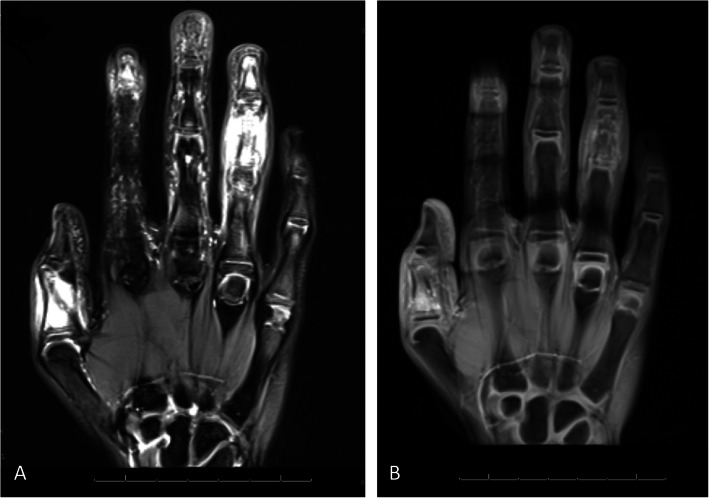
Right hand MRI **a**. Coronal proton density fat saturation sequences of the right hand shows increased water signal involving multiple phalanges, but most prominently the first proximal phalanx and fourth middle phalanx. Abnormal marrow signal is associated with periosteal lifting and soft tissue edema. **b**. Coronal T1 post-contrast with fat saturation shows contrast enhancement of the marrow space and soft tissue. Note the absence of joint effusions and synovial enhancement

Hand radiographs showed marrow and cortical erosive change with periosteal reaction and overlying soft tissue swelling of the involved phalanges (Fig. 2). MRI of the right hand revealed signal abnormalities with surrounding soft tissue edema, but without arthritis, in these areas (Fig. 3). These findings prompted concern for chronic noninfectious osteomyelitis (CNO) or an alternate noninfectious inflammatory entity, such as Langerhans Cell Histiocytosis (LCH). Subsequent whole body imaging did not identify any other areas of involvement. The isolation of the lesions to the hands was felt to be atypical for CNO and LCH, particularly without evidence of systemic inflammation to further suggest such diagnoses. Therefore, further evaluations for other etiologies of finger swelling with bony changes were pursued.

Laboratory data were significant for normal complete blood count, iron studies, erythrocyte sedimentation rate, C-reactive protein, and urinalysis. Immunologic serologies were similarly negative, including antinuclear antibody, Rheumatoid Factor, Human Leukocyte Antigen-B27, Celiac antibodies, and Lyme antibodies. Thyroid stimulating hormone and free thyroxine were only very slightly increased and decreased, respectively, but primary hypothyroidism was not thought to be causative of the marked failure to thrive. Hepatic function was intact, with normal albumin level and coagulation studies, but low prealbumin of 18.6 mg/dL (reference range 20–26 mg/dL) was suggestive of a nutritional deficit. Further investigation of vitamin and mineral status showed normal zinc, folate, and vitamin B6 levels. However, vitamin C was strikingly low at 5 μmol/L (reference range 23–114 μmol/L), as was vitamin D 25-OH at 12.2 ng/mL (normal > 30 ng/mL).

The diagnosis of scurvy was made on the basis of a severely low vitamin C level. Although scurvy bone lesions are typically described in the lower extremities, since the analogous lesions were present in the hands this was thought to be consistent with vitamin C deficiency. These vitamin deficiencies were attributed to restricted intake due to anorexia nervosa, and treatment was initiated with ascorbic acid, cholecalciferol, and liquid meal supplements. The ascorbic acid regimen consisted of 100 mg 3 times per day orally for 7 days, followed by 100 mg daily as maintenance; cholecalciferol dosing was 2000 U orally daily. Within 3 weeks of starting therapy, vitamin levels normalized and the digital pain and swelling resolved.

## Discussion

Humans are unable to produce ascorbic acid and, therefore, rely on exogenous intake to meet their physiologic needs. Its role as a cofactor in a variety of hydroxylation reactions makes vitamin C essential to processes such as collagen synthesis, hematopoiesis, angiogenesis, neural myelination, and iron and glucose metabolism [2, 6, 7]. Perhaps the most clinically well described consequences of deficiency are mucocutaneous hemorrhage, dermatitis, and bone pain. As mice are not dependent on dietary intake of vitamin C, targeted gene deletion mouse models carrying defects in vitamin C synthesis pathways serve as a system in which to study the scurvy syndrome; both human and murine scurvy symptoms correct with dietary repletion [7, 8].

The United States National Health and Nutrition Examination Survey data from 2003 to 2004 demonstrated that less than 4% of adolescents are vitamin C deficient [9]. The frequency of pediatric scurvy in published case series and individual reports suggests that neurodevelopmental disorders, such as autism spectrum disorder, are among the most common causes of restricted intake of vitamin C-containing foods [10]. Restricted intake secondary to eating disorders is known, as well, and was the cause of vitamin C deficiency in our adolescent male patient [11]. The bone pain described in most cases is typically diffuse in the lower extremities, with very young children refusing to ambulate, and imaging findings in corresponding locations. To our knowledge, this is the first report of scurvy bone disease manifesting exclusively in the hands.

Beyond a low serum vitamin C level, skeletal imaging is an important component of the scurvy work up and diagnosis. The most suggestive features are often easier to identify on plain film rather than MRI, whose findings of marrow edema with abnormal periosteal and soft tissue signal can be fairly nonspecific and may prompt a broad work up for inflammatory conditions, infection, and metabolic disorders [5]. Our patient exhibited some of the classic scurvy x-ray signs, which can include cortical thinning, erosions with joint space narrowing, periosteal reaction, and a “scorbutic zone” of a lucent metaphyseal band underlying a dense zone of provisional calcification [12, 13]. With the exception of the scorbutic zone, these features can be present in inflammatory arthropathies, therefore, it is imperative to interpret imaging findings in the context of the larger clinical picture, which in this case was most suggestive of scurvy. The bone resorptive effects of vitamin C deficiency result from both impaired osteoblast function and increased osteoclast proliferation, the latter being a function of elevated Receptor Activator of Nuclear Factor-κB Ligand (RANKL) expression in bone marrow [14]. RANKL, a member of the Tumor Necrosis Factor (TNF) superfamily, promotes osteoclast differentiation, activity, and survival, and is implicated in the bone and joint destruction characteristic of rheumatoid arthritis [15]. This pathway suggests that abnormally low levels of ascorbic acid could invoke pathologic bone turnover that mimics inflammatory bone disease, thereby accounting for the MRI changes.

As our patient was not systemically inflamed or cytopenic, the concern for malignancy or infection was low, thus inflammatory and metabolic etiologies were pursued until the diagnosis was established on the basis of the abnormal vitamin C level, despite the absence of other classic scurvy stigmata, such as gingival bleeding or purpuric skin lesions. It was noted that the low vitamin D level may have contributed to the patient’s symptoms, but there was not the typically associated osteopenia, nor would hypovitaminosis D alone have explained the soft tissue edema or periosteal reaction seen on MRI.

## Conclusions

We report a case of scurvy in a patient with anorexia nervosa mimicking inflammatory bone disease of the hand, that improved with vitamin C repletion. Most vitamin C deficiency-related skeletal changes manifest in the lower extremities and, to our knowledge, this is the first description of such a syndrome presenting in the fingers. As exam and imaging findings are similar to inflammatory bone processes, such as osteomyelitis, there must be a high index of suspicion for scurvy in children who have bone pain in the setting of limited oral intake or impaired enteral absorption, irrespective of location of the lesions.

## Data Availability

Not applicable.

## Abbreviations

MRI: Magnetic resonance imaging
CNO: Chronic noninfectious osteomyelitis
LCH: Langerhans cell histiocytosis
RANKL: Receptor activator of nuclear factor-κB ligand
TNF: Tumor necrosis factor
